# A multiplex PCR assay for the simultaneous detection of *Taenia hydatigena*, *T. multiceps*, *T. pisiformis*, and *Dipylidium caninum* infections

**DOI:** 10.1186/s12879-019-4512-3

**Published:** 2019-10-16

**Authors:** Guo-Qiang Zhu, Li Li, John Asekhaen Ohiolei, Yan-Tao Wu, Wen-Hui Li, Nian-Zhang Zhang, Bao-Quan Fu, Hong-Bin Yan, Wan-Zhong Jia

**Affiliations:** 1 State Key Laboratory of Veterinary Etiological Biology/ Key Laboratory of Veterinary Parasitology of Gansu Province/ Lanzhou Veterinary Research Institute, CAAS, Lanzhou, 730046 Gansu Province People’s Republic of China; 2 Jiangsu Co-innovation Center for Prevention and Control of Important Animal Infectious Disease, Yangzhou, 225009 Jiangsu Province, People’s Republic of China

**Keywords:** Multiplex PCR, Canids, Tapeworms, Mitochondrial DNA

## Abstract

**Background:**

*Taenia hydatigena*, *T. multiceps*, *T. pisiformis*, and *Dipylidium caninum* are four common large and medium-sized tapeworms parasitizing the small intestine of dogs and other canids. These parasites cause serious impact on the health and development of livestock. However, there are, so far, no commercially available molecular diagnostic kits capable of simultaneously detecting all four parasites in dogs. The aim of the study was therefore to develop a multiplex PCR assay that will accurately detect all four cestode infections in one reaction.

**Methods:**

Specific primers for a multiplex PCR were designed based on corresponding mitochondrial genome sequences, and its detection limit was assessed by serial dilutions of the genomic DNAs of tapeworms examined. Furthermore, field samples of dog feces were tested using the developed assay.

**Results:**

A multiplex polymerase chain reaction (PCR) assay was developed based on mitochondrial DNA (*mt*DNA) that accurately and simultaneously identify four cestode species in one reaction using specific fragment sizes of 592, 385, 283, and 190 bp for *T. hydatigena*, *T. multiceps*, *T. pisiformis*, and *D. caninum*, respectively. The lowest DNA concentration detected was 1 ng for *T. hydatigena*, *T. multiceps* and *T. pisiformis*, and 0.1 ng for *D. caninum* in a 25 μl reaction system. This assay offers high potential for the rapid detection of these four tapeworms in host feces simultaneously.

**Conclusions:**

This study provides an efficient tool for the simultaneous detection of *T. hydatigena*, *T. multiceps*, *T. pisiformis*, and *D. caninum*. The assay will be potentially useful in epidemiological studies, diagnosis, and treatment of these four cestodes infections during prevention and control program.

## Background

Historically, cestodes are parasites that have caused serious damage to wild and domestic animals as well as human health since antiquity, resulting in economic losses by affecting food safety, livestock production, and serious public health consequences [1, 2]. Some cestode infection such as those caused by *Echinococcus* and *Taenia* are widespread and of significant public health concerns with challenges of prevention and control [3–8]. For example, a recent survey report from Tanzania has shown that the infection rates of cysticercosis in goats and sheep could reach as high as 61.1 and 42.2%, respectively [6]; the prevalence rates of alveolar echinococcosis (AE) cases of childhood as high as 12.1 (Tehetu) and 11.8% (Moba) in Dari County, China [9].

*Taenia hydatigena*, *T. multiceps*, *T. pisiformis*, and *D. caninum* are four common parasites that parasitize the small intestine of dogs with their larvae causing cysticercosis tenuicollis, coenurosis, cysticercosis pisiformis and dipylidiasis in intermediate hosts, respectively. Cats and foxes also can act as definitive hosts of *D. caninum* [10]. In some cases, these parasites have resulted in serious medical concerns in different parts of the world due to their zoonotic potential in humans [10, 15–18], while seriously impacting the health and development of animals/livestock [11–14]. For instance, a recent study indicated that 9.1% of *D. caninum* infection rates occurred in dogs in Europe [19] and 6.3% of red foxes were found to be infected with *T. multiceps* [20]; the overall infection rates for *T. hydatigena* were 61.1% in goats and 42.2% in sheep in a Malambo (a village) slaughter slab in Tanzania [6]; and 8.3% *T. hydatigena* positive dogs and foxes in Australia [21]. Therefore, specific and effective detection methods are highly crucial for the identification, prevention and control of these parasitic infections [22].

So far, there are no commercially available molecular diagnostic kits for the simultaneous detection of multiple infections by all four cestodes in a host. Although enzyme-linked immunosorbent assay (ELISA) is being used for the diagnosis of *T. hydatigena*, *T. multiceps*, and *T. pisiformis* infections, challenges of misdiagnosis as a result of false-positive and false-negative remains a major issue [23–25]. The multiplex PCR assay permits the simultaneous and accurate detection of multiple parasites [26, 27], and the mitochondrial (*mt*) DNAs are one of the important multiplex PCR target molecules owing to their particular characteristics of nucleotide variation and high polymorphism [28, 29]. Consequently, for the first time, we developed a multiplex PCR assay to detect and discriminate *T*. *hydatigena*, *T*. *multiceps*, *T*. *pisiformis* and *D*. *caninum* infections, which will be important for the epidemiology, diagnoses, and control of cestode infections.

## Methods

### Origin of specimens and DNA extraction

Samples of adult parasites and dog feces were obtained from the Animal Center of State Key Laboratory of Veterinary Etiological Biology, Lanzhou Veterinary Research Institute, CAAS, People’s Republic of China, and stored at − 80 °C until DNA extraction. Genomic DNAs were extracted from adult parasites using DNeasy® Blood and Tissue Kit (QIAGEN, Hilden, Germany) and dog feces (180–220 mg) using QIAamp® DNA Stool Mini Kit (QIAGEN, Hilden, Germany), DNA elution was achieved with 20 μl of elution buffer. DNA concentration and purity were determined using a spectrophotometer (TECAN, Tecan’ Infinite® 200 PRO NanoQuant) and stored at − 20 °C until use [28].

### Primer design

Based on the alignment of the *mt*DNAs of *T. hydatigena*, *T. multiceps*, *T. pisiformis* and *D. caninum* with online tool ClustalW (http://www.simgene.com/ClustalW), multiplex PCR primers were designed by targeting conserved sequences flanking variable regions with the help of Oligo primer software. Each set of primer pairs were checked for specificity using the NCBI database online tool Nucleotide BLAST (https://blast.ncbi.nlm.nih.gov/Blast.cgi). Details of primer pairs are presented in Table 1.

**Table 1:** Forward and reverse primers used in the multiplex PCR for *Taenia hydatigena*, *T. multiceps*, *T. pisiformis*, and *Dipylidium caninum*

Species	Primer names and sequences 5′-3’	Expected amplicon size (bp)	The location of primers in the *mt*DNA	Accession no. of *mt*DNAs in NCBI GenBank
*T. hydatigena*	Th-F: AGTTCCATATTATTTACAGTTTTGTTATTAC	592	12,446	NC_012896
	Th-R: TAACATAATACTTGAAGACACCCCCA		13,038	
*T. multiceps*	Tm-F: GTTGTTGATGTGGCTTAAGTTTTTGTGT	385	10,902	NC_012894
	Tm-R: TCTATAAAATAAACACATACACAACAATCCT		11,287	
*T. pisiformis*	Tp-F: TGTGGGAAGGTTTAGGTGAATCAT	283	200	NC_013844
	Tp-R: GTTAACATCAATATCTTCTAGCTCTGACACT		483	
*D. caninum*	Dc-F: CTATTGATTGCGTTTATTGTTTTGTGT	190	8594	MG587892
	Dc-R: GAAAAGAAATCAAATACAGTTAAACGGT		8784	

Annotation: F, forward primer; R, reverse primer; bp, base pairs

### Multiplex PCR assay

Multiplex PCR reaction parameters were optimized and the reaction was carried out in a final reaction volume of 25 μl containing 0.1 μg genomic DNA, 4 μl optimal primers with each primer (10 μM) contributing 0.5 μl (Th-F/R for *T. hydatigena*, Tm-F/R for *T. multiceps*, Tp-F/R for *T. pisiformis* and Dc-F/R for *D. caninum*), and 12.5 μl Premix *Taq*™ (5 U/ml) (Takara Bio, Japan). Fragments were amplified using the following optimized thermal cycling conditions: 95 °C/5 min for initial denaturation; 30 cycles of 94 °C/30 s for denaturation, 55 °C/30 s for annealing, 72 °C/40 s for extension; 72 °C/10 min for final extension; and amplification products were stored at 4 °C until they were visualized.

### Identification of PCR products

PCR products (8 μl) were visualized by electrophoresis in 2.0% (w/v) agarose gels that were pre-stained with ethidium bromide (EB) and viewed under UV light (BIO-RAD, Molecular Imager® chemiDoc™ XRS+ with image lab™ software). The electrophoretic buffer solution was 1 × TAE buffer [30].

### Specificity and limit of the assay

The specificity of the multiplex PCR was verified using genomic DNAs of other common tapeworms (*E. canadendsis* (G7), *E. granulosus*, *E. multilocularis* and *E. shiquicus*) inhabiting the small intestine of canids. Furthermore, the lowest detected DNA was verified using serial dilutions of genomic DNA of each parasite in nuclease-free water, and the final DNA concentration for each parasite in a 25 μl reaction system was 10, 1.0, 1.0 × 10^− 1^, 1.0 × 10^− 2^, 1.0 × 10^− 3^, 1.0 × 10^− 4^ ng, respectively.

### Field application of the multiplex PCR

The usefulness of the newly developed assay was verified using genomic DNAs from dog feces as templates. The multiplex PCR assay was carried out in a final reaction mixture of 25 μl, containing 2 μl templates, 4 μl optimal primers with 0.5 μl from each forward and reverse primer (Th-F/R for *T. hydatigena*, Tm-F/R for *T. multiceps*, Tp-F/R for *T. pisiformis* and Dc-F/R for *D. caninum*), and 12.5 μl Premix *Taq*™ (Takara Bio, Japan), followed by thermal cycling conditions and visualization process mentioned above.

## Results

### Specificity

**Fig. 1:**
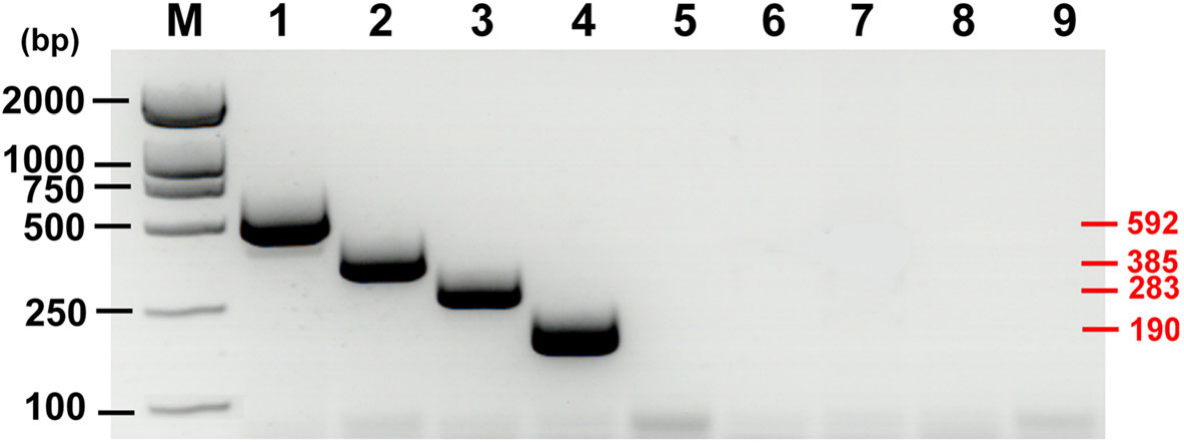
Development of the multiplex PCR specific assay for four pathogenic dogs parasites. Lane 1–8: The products of PCR amplification using the genomes of *Taenia hydatigena*, *T. multiceps*, *T. pisiformis*, *Dipylidium caninum*, *Echinococcus canadensis*, *E. granulosus*, *E. multilocularis* and *E. shiquicus* as templates, respectively; Lane 9: negative control; Lane M: 2000 bp DNA standard marker

According to the location of designed primers, the expected size of multiplex PCR amplicons for each species was 592 bp (*T*. *hydatigena*), 385 bp (*T*. *multiceps*), 283 bp (*T*. *pisiformis*) and 190 bp (*D*. *caninum*), respectively. As expected, all PCR products were of exact band sizes, and when DNA templates from other tapeworms were involved in the multiplex PCR assay, no PCR products were produced (Fig. 1).

**Fig. 2:**
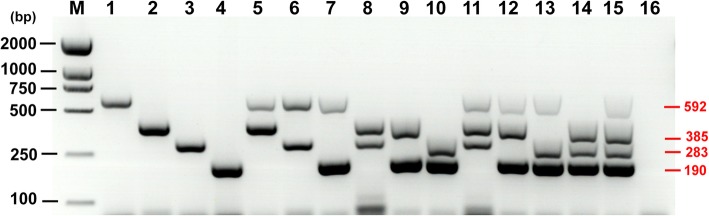
The specificity analysis of the 4 sets of primers using various combined target templates. Lanes 1–4: PCR amplification result of *Taenia hydatigena*, *T. multiceps*, *T. pisiformis* and *Dipylidium caninum* as single template, respectively; Lanes 5–10: PCR amplification result of Th + Tm, Th + Tp, Th + Dc, Tm + Tp, Tm + Dc and Tp + Dc as dual templates, respectively; Lanes 11–14: PCR amplification result of Th + Tm + Tp, Th + Tm + Dc, Th + Tp + Dc and Tm + Tp + Dc as triple templates, respectively; Lane 15: PCR amplification result of Th + Tm + Tp + Dc as quadruple templates; Lane 16: negative control; Lane M: 2000 bp DNA standard marker

The multiplex PCR products of mixed templates of the four parasites are shown in Fig. 2. The products containing two DNA bands (592 and 385 bp, 592 and 283 bp, 592 and 190 bp, 385 and 283 bp, 385 and 190 bp, 283 and 190 bp) were amplified with mixed DNA templates of *T. hydatigena* and *T. multiceps*, *T. hydatigena* and *T. pisiformis*, *T. hydatigena* and *D. caninum*, *T. multiceps* and *T. pisiformis*, *T. multiceps* and *D. caninum*, *T. pisiformis* and *D. caninum*, respectively. Three DNA bands (592, 385 and 283 bp; 592, 385 and 190 bp; 385, 283 and 190 bp) were amplified with mixed DNA templates of *T. hydatigena*, *T. multiceps* and *T. pisiformis*; *T. hydatigena*, *T. multiceps* and *D. caninum*; *T. multiceps*, *T. pisiformis* and *D. caninum*. Four DNA bands (592, 385, 283 and 190 bp) were amplified with mixed DNA templates of *T. hydatigena*, *T. multiceps*, *T. pisiformis* and *D. caninum* (Fig. 2).

### Detection minimum limit

**Fig. 3:**
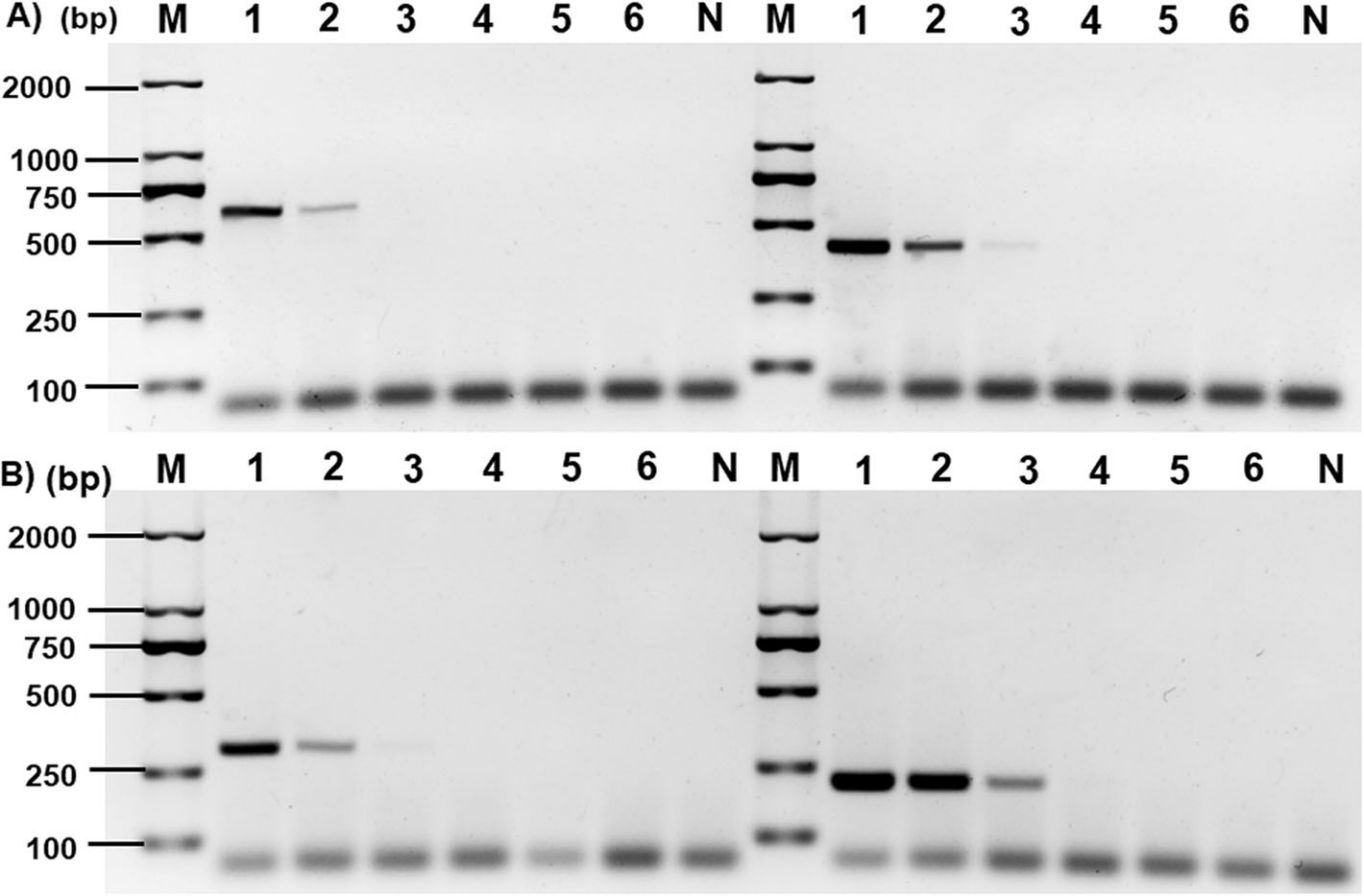
The multiplex PCR sensitivity assay for four pathogenic dogs parasites. Lanes 1–6: the concentrations of serial dilutions differ templets are 10, 1.0, 1.0 × 10^− 1^, 1.0 × 10^− 2^, 1.0 × 10^− 3^, 1.0 × 10^− 4^ ng, respectively; Lane M: 2000 bp DNA standard marker; Lane N is negative control. **a):** This picture showing the amplicons of 592 and 385 bp produced in a 25 μl PCR system with differ DNA concentrations of *Taenia hydatigena* (left) and *T. multiceps* (right) templates, respectively; **b):** This Picture showing the amplicons of 283 and 190 bp produced in a 25 μl PCR system with differ DAN concentrations of *T. pisiformis* (left) and *Dipylidium caninum* (right) templates, respectively

The minimum detection limit was determined by different DNA gradients with 10, 1.0, 1.0 × 10^− 1^, 1.0 × 10^− 2^, 1.0 × 10^− 3^, 1.0 × 10^− 4^ ng, respectively. The results indicated that the lowest limit for the DNA detection was 1 ng for *T*. *hydatigena*, *T*. *multiceps* or *T*. *pisiformis* and 0.1 ng for *D*. *caninum*, respectively (Fig. 3).

### Evaluation of field samples

**Fig. 4:**
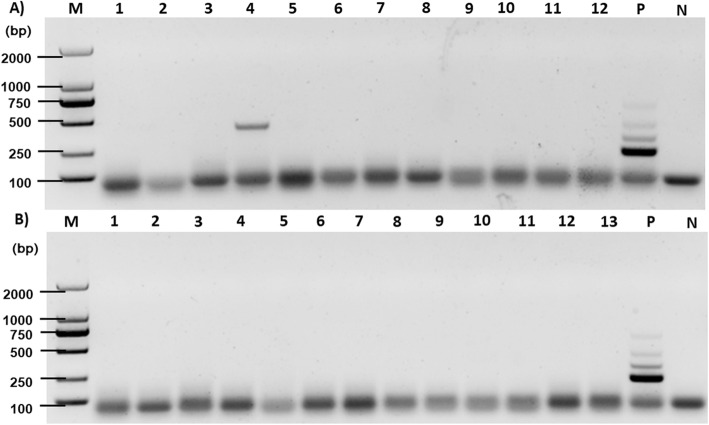
The PCR results of DNA sample from dog feces. **a** and **b** represent the results of two sets of PCR experiments for dog feces**.** Lane M: 2000 bp DNA standard marker; Lanes 1–12: DNA sample of dog feces; Lane P: Positive control with genomic DNA from adult parasites of representative tapeworms from dogs; Lane N: negative control

In the field application of this multiplex PCR assay, a total of 25 dog feces were tested. One sample tested positive with a fragment size of about 385 bp identified as *T. multiceps* infection (Fig. 4).

## Discussion

Until now, due to the lack of accurate and simultaneous detection methods in a single reaction for these four cestode parasites (*T. hydatigena*, *T. multiceps*, *T. pisiformis and D. caninum*), it is noteworthy that traditional detection and diagnosis mostly relies on post-mortem examination by experts, which is laborious and time-consuming [13, 31] coupled with the challenge of misdiagnosis [10, 32]. Although the diagnosis can also be achieved with immunodiagnostic assays such as antigen enzyme-linked immunosorbent assay (Ag-ELISA), cross-reaction with other *Taenia* spp. sharing similar antigenic properties has also been a challenge [23]. In this study, we developed a multiplex assay which is sensitive to discriminate and diagnose four cestode parasites simultaneously in a single reaction compare to the conventional PCR method (requiring only a pair of primers in a reaction system). The specificity analysis showed that no cross-reactivity was observed with other tapeworms inhabiting the small intestine as well as with each other, and the minimum detected DNA ranged from 0.1 to 1 ng, low enough to produce results in case of low DNA yield.

Two obligate mammalian hosts are essential for the transmission of some cestode parasites including *T. hydatigena*, *T. multiceps* and *T. pisiformis*, which is dependent on prey-predator associations of intermediate hosts (including humans) and definitive hosts [33]. Infections by these parasites often lead to serious damage in intermediate hosts than in definitive hosts where infections are subclinical [34–36]. Therefore, effective control measures are often targeted at the definitive hosts that are capable of transmitting and maintaining the infection within an environment. Similarly, this is also the case for *Echinococcus* spp. infections which are commonly observed in dogs and other canids in China [37, 38]. However, the recent establishment of a multiplex PCR method for the detection of multiple infections with *E. granulosus* sensu stricto, *E. multilocularis* and *E. shiquicus* [28], has provided a rapid, sensitive, and cost-effective method in discriminating these infections. Also, the establishment of a detection method that is accurate and inexpensive for the discrimination of *T. hydatigena*, *T. multiceps*, *T. pisiformis* and *D. caninum* infections in definitive hosts is a step further in achieving prevention and control.

Mitochondrial genes are amongst the most popular molecular markers that have been widely used in molecular ecology, population genetics, and diagnoses of parasitic organisms [26, 39]. Therefore, we explored the *mt* genes as molecular markers to develop a multiplex PCR system for the synchronous detection of the four tapeworm infections. However, some challenges were confronted with sample collection and fecal DNA extraction. For example, poor DNA yield, which could be due to a number of inhibitors and the complex presence of other organisms in the feces, the of feces (fresh or old) and the uneven distribution of parasite eggs.

Nonetheless, with the continuous advancement in biomedical/life sciences, alternative molecular markers such as microRNAs (miRNAs) [40], microsatellite DNAs [41] and improved DNA extraction methods may provide further opportunity for the development of more detection and diagnostic methods for epidemiological investigations.

We admit that the current study had a limitation of appropriately assessing the sensitivity of this assay. However, we recommended that, prior to DNA extraction, conventional flotation/sedimentation technique should be used to concentrate cestode eggs from feces in order to increase the sensitivity. Also, regarding the presence of other cestodes of canids such as *T. ovis*, *T crassiceps*, *T. serialis* and *T. gaigeri* which were not assessed in this study, application of this test will be limited to areas where the four tapeworms *T. hydatigena*, *T. multiceps*, *T. pisiformis* and *D. caninum* are prevalent. Nevertheless, the design principles or skills of multiplex PCR primers and methods in this study and others can be followed or adopted in other areas where other taenids co-exist in canids. Moreover, standard parasitological methods should be used simultaneously to differentiate the worms.

## Conclusion

The multiplex PCR assay is an efficient tool for the detection and simultaneous diagnosis of *T. hydatigena*, *T. multiceps*, *T. pisiformis*, and *D. caninum* tapeworms from DNA sample, the lowest limit of detectable DNA was 1 ng for *T. hydatigena*, *T. multiceps*, and *T. pisiformis*, and 0.1 ng for *D. caninum*, respectively. Consequently, this assay will be potentially useful in epidemiological studies, diagnosis, and treatment of taeniasis or cestode infections during prevention and control program.

## Data Availability

The datasets used and/or analyzed during the current study are available from the corresponding author on reasonable request.

## Abbreviations

ELISA: Enzyme-linked immunosorbent assay
*mt*: mitochondrial; bp: base pair
PCR: Polymerase chain reaction
